# Neuregulin-1 as a Context-Dependent Regulator of Neuroinflammation and Neural Repair: Mechanisms, Disease Relevance, and Therapeutic Challenges

**DOI:** 10.3390/cells15181705

**Published:** 2026-09-19

**Authors:** Gregory D. Ford, Christopher McGinley, Oluwafayokemi Oyolola, Oyinkansola Adeyemi, Monique C. Surles-Zeigler, Shokofeh Rahimpour, Byron D. Ford

**Affiliations:** 1 Department of Anatomy, Howard University College of Medicine, Washington, DC 20059, USA; gregory.ford@howard.edu (G.D.F.); christopher.mcginley@bison.howard.edu (C.M.); oluwafayokemi.oyolo@bison.howard.edu (O.O.); oyin.adeyemi@howard.edu (O.A.); monique.surleszeigl@howard.edu (M.C.S.-Z.); shokofeh.rahimpour@howard.edu (S.R.); 2 Division of Biomedical Sciences, University of California-Riverside School of Medicine, Riverside, CA 92521, USA

**Keywords:** ErbB4, ErbB2, microglia, astrocytes, NF-κB, alpha7 nicotinic acetylcholine receptor, blood–brain barrier, neuroinflammation, remyelination

## Abstract

**Highlights:**

**What are the main findings?**

- NRG1 emerges as a context-dependent regulator of neuroinflammation that coordinates microglial, astrocytic, oligodendroglial, neuronal, and neurovascular responses through ErbB receptor signaling rather than functioning as a uniformly anti-inflammatory molecule.
- Across multiple preclinical models, NRG1 consistently demonstrates neuroprotective and immunomodulatory properties, although the strength of evidence and underlying mechanisms vary substantially by disease model.

**What are the implications of the main findings?**

- Future NRG1 therapeutics should emphasize receptor-selective, isoform-specific, and anatomically targeted approaches to maximize neuroprotection.
- A deeper understanding of NRG1 isoform biology, receptor heterodimer composition, and cell-type-specific signaling networks will enable precision neuroimmune therapies capable of simultaneously limiting inflammation while promoting neural repair.

**Abstract:**

Neuroinflammation is a coordinated response to central nervous system injury and disease involving resident glia, neurons, the neurovascular unit, and infiltrating immune cells. Although transient inflammatory signaling supports host defense, debris clearance, and repair, persistent activation contributes to synaptic dysfunction, demyelination, blood–brain barrier disruption, and neuronal loss. Neuregulin-1 (NRG1), a pleiotropic epidermal growth factor family ligand, has emerged as a potential regulator of this balance. Through ErbB receptor complexes, particularly ErbB4-containing dimers, NRG1 influences neural development, myelination, synaptic function, cell survival, and inflammatory signaling. Experimental evidence indicates that NRG1 can restrain NF-κB-dependent transcription, alter microglial activation states, enhance alpha7 nicotinic acetylcholine receptor-associated anti-inflammatory signaling, support oligodendroglial lineage cells, and stabilize neurovascular integrity. However, these actions are context-dependent; in spinal nociceptive circuits, ErbB2-linked signaling can promote microglial activation and pain hypersensitivity. This review examines NRG1 isoform biology, ErbB receptor architecture, cellular targets, and disease-specific evidence across demyelinating disease, stroke, traumatic brain injury, neurodegeneration, cerebral malaria, sickle cell disease, and neuropathic pain. Translation will require isoform-specific, receptor-biased, and anatomically targeted approaches supported by rigorous in vivo validation and verified biomarkers.

## 1. Introduction

Neuroinflammation is a highly coordinated and evolutionarily conserved response that protects the central nervous system (CNS) from infection, trauma, ischemia, and neurodegenerative insults [1,2,3,4]. Under normal physiological conditions, interactions among neurons, glia, vascular cells, and infiltrating immune cells maintain tissue homeostasis by eliminating cellular debris, limiting pathogen spread, and promoting repair. However, when inflammatory responses become excessive, persistent, or dysregulated, they contribute to blood–brain barrier (BBB) dysfunction, synaptic loss, axonal degeneration, demyelination, and progressive neuronal death. Chronic neuroinflammation is now recognized as a common pathogenic mechanism underlying numerous neurological disorders, including ischemic stroke, traumatic brain injury (TBI), multiple sclerosis (MS), Alzheimer’s disease (AD), Parkinson’s disease (PD), cerebral malaria, and sickle cell disease-associated neurological complications [5]. Despite considerable advances in understanding the molecular and cellular mechanisms driving neuroinflammation, there is a need to identify effective therapies that promote inflammatory resolution while preserving protective immune functions.

Traditional therapeutic strategies have primarily focused on suppressing individual inflammatory mediators or blocking specific immune pathways. While these approaches have demonstrated efficacy in preclinical models, they have generally failed to produce meaningful clinical benefits across diverse neurological diseases. These disappointing outcomes highlight the complexity of CNS immune responses and suggest that successful neuroprotective therapies will require coordinated modulation of multiple interacting cell types and signaling pathways, rather than indiscriminate suppression of inflammation. Increasing evidence indicates that endogenous repair pathways capable of simultaneously regulating immune responses, maintaining neurovascular integrity, promoting remyelination, and supporting neuronal survival may represent a more effective therapeutic strategy.

### NRG1 Is a Compelling Neuroimmune Tuning Axis

Neuregulin-1 (NRG1), a member of the epidermal growth factor (EGF) family of signaling proteins, has emerged as one such multifunctional regulator of CNS homeostasis. Originally characterized for its essential roles in embryonic development, cardiac morphogenesis, Schwann cell maturation, and synapse formation, NRG1 is now recognized as a pleiotropic signaling molecule that continues to regulate neural function throughout adulthood [6,7,8,9,10].

NRG1 exerts its biological effects primarily through activation of ErbB receptor tyrosine kinases, particularly ErbB3 and ErbB4, initiating downstream signaling cascades that include phosphatidylinositol-3 kinase (PI3K)/Akt, mitogen-activated protein kinase (MAPK)/extracellular signal-regulated kinase (ERK), Janus kinase (JAK)/signal transducer and activator of transcription (STAT), AMP-activated protein kinase (AMPK), cyclic AMP response element-binding protein (CREB), and nuclear factor-kappa B (NF-κB) [11,12,13,14,15,16,17,18,19,20]. These interconnected pathways regulate diverse biological processes, including cell survival, inflammation, oxidative stress, mitochondrial function, synaptic plasticity, oligodendrocyte maturation, and tissue repair.

Recent investigations have substantially expanded our understanding of NRG1 biology, revealing immunomodulatory effects that extend far beyond classical neurotrophic functions. Experimental studies indicate that NRG1 can influence multiple cellular components of the neuroimmune system, including microglia, astrocytes, oligodendrocyte lineage cells, neurons, and endothelial cells [9,12,17,19,21,22,23,24,25]. Through these interactions, NRG1 has been associated with changes in inflammatory cytokine production, glial activation, BBB integrity, leukocyte infiltration, remyelination, and neuronal repair. The strength of evidence for each effect differs across experimental systems. Collectively, these findings support viewing NRG1 as a context-dependent regulator whose biological effects vary according to receptor composition, cellular targets, anatomical location, disease stage, and local microenvironment.

This concept of context-dependent signaling has become increasingly important in understanding the diverse biological actions of NRG1. In several experimental models of brain injury, ErbB4-containing receptor complexes have been associated with reduced inflammatory signaling, improved BBB stability, suppression of NF-kB-dependent responses, oligodendrocyte differentiation, and neuronal survival [12,19,23,26,27,28,29]. In contrast, within spinal nociceptive circuits, NRG1 signaling through ErbB2-containing receptor complexes can activate microglia, stimulate MEK/ERK signaling, increase pro-inflammatory mediators, and contribute to central sensitization and neuropathic pain [30]. These divergent observations suggest that receptor heterodimer composition, cellular context, and regional neuroanatomy are important determinants of outcome.

Growing evidence from experimental models further supports a broad role for NRG1 in regulating neuroimmune responses across diverse neurological disorders. In models of ischemic stroke, NRG1 reduces infarct volume, preserves BBB integrity, attenuates microglial activation, suppresses NF-κB signaling, promotes oligodendrocyte survival, and improves long-term neurological recovery [9,10,12,19,31,32,33,34]. Similar immunomodulatory and neuroprotective effects have been reported in TBI, MS, AD, PD, cerebral malaria, and sickle cell disease. However, the relative contributions of different signaling pathways vary among disease states. Together, these observations suggest that NRG1 represents a conserved regulator of neuroimmune homeostasis that coordinates inflammatory resolution with tissue repair across multiple forms of CNS injury.

Despite these advances, several critical questions remain unresolved. The biological functions of individual NRG1 isoforms remain incompletely understood, receptor-selective signaling mechanisms have yet to be fully elucidated, and the influence of aging, sex, disease stage, and comorbidities on therapeutic responsiveness remains largely unexplored. Furthermore, successful clinical translation will require optimization of drug delivery strategies, pharmacokinetics, receptor specificity, biomarker development, and long-term safety while minimizing unwanted activation of signaling pathways associated with adverse effects.

In this review, we synthesize current knowledge regarding the biology of NRG1 and its emerging role as a context-dependent regulator of neuroimmune homeostasis. We examine the molecular mechanisms by which NRG1 influences microglia, astrocytes, oligodendrocytes, neurons, and the neurovascular unit, evaluate evidence supporting its therapeutic potential across multiple neurological diseases, and discuss the opportunities and challenges associated with clinical translation. We propose that NRG1 should be viewed not simply as a neurotrophic growth factor or anti-inflammatory cytokine, but as a context-dependent regulator of neuroinflammation whose therapeutic potential lies in the precise modulation of receptor-, cell-, and region-specific signaling pathways that coordinate inflammatory resolution with neural protection and repair. This conceptual framework provides a unifying perspective for understanding the diverse biological actions of NRG1 and identifies promising directions for the development of next-generation neuroimmune therapeutics.

## 2. NRG1 Biology and Signaling Architecture

NRG1 is a multifunctional growth factor that regulates numerous developmental and homeostatic processes within the central and peripheral nervous systems [9,35,36,37]. Initially identified as a ligand for ErbB receptor tyrosine kinases, NRG1 is now recognized as a highly complex signaling molecule whose biological activities extend beyond neuronal development to include regulation of neuroinflammation and neurovascular integrity, as well as myelination, synaptic plasticity, and tissue repair. Unlike classical cytokines or neurotrophic factors that exert relatively uniform biological effects, NRG1 signaling is remarkably diverse, with outcomes determined by ligand isoform, receptor composition, cellular target, anatomical location, and disease context. This signaling complexity underlies both the broad therapeutic potential and the translational challenges associated with targeting the NRG1–ErbB signaling axis.

### 2.1. NRG1 Isoform Diversity and Structural Organization

The human NRG1 gene, located on chromosome 8p12, is one of the most structurally complex genes in the mammalian genome [6,38,39]. Extensive alternative promoter usage and alternative splicing generate more than 30 transcript variants that encode multiple protein isoforms with distinct structural domains, expression patterns, and biological functions. Based on differences in their N-terminal regions, NRG1 isoforms have traditionally been classified into at least six major types (Types I–VI), although additional variants continue to be identified [9,35,36,37]. Despite their structural diversity, all biologically active NRG1 isoforms contain a conserved EGF-like domain, which is both necessary and sufficient for binding and activating ErbB receptors. Outside this conserved region, isoforms differ substantially in their immunoglobulin-like domains, cysteine-rich domains, transmembrane regions, intracellular tails, and subcellular localization, resulting in distinct signaling mechanisms and tissue-specific functions.

In addition to structural classification, NRG1 isoforms are further distinguished by the sequence of their EGF-like domains, which are broadly categorized as α (alpha) and β (beta) variants. These isoforms differ by several amino acid substitutions within the receptor-binding EGF domain that markedly influence receptor affinity and biological potency. In general, NRG1β isoforms exhibit substantially higher affinity for ErbB3 and ErbB4 receptors and produce more robust receptor phosphorylation and downstream signaling than corresponding α isoforms, making β isoforms the predominant mediators of neuronal development, myelination, synaptic plasticity, and neuroprotection [7,37,40,41,42]. By contrast, NRG1α isoforms display lower receptor affinity and weaker mitogenic activity, and although they are broadly expressed in peripheral tissues and during development, their physiological roles within the adult CNS remain comparatively less well understood. Most experimental studies investigating neuroprotection and neuroinflammation have therefore utilized recombinant NRG1β1, particularly the soluble EGF-like domain, which has become the canonical experimental ligand for investigating NRG1 signaling.

Type I NRG1 is broadly expressed throughout the nervous system and is the predominant soluble β isoform implicated in neuroprotection and inflammatory regulation. Following proteolytic cleavage, Type I NRG1 is released into the extracellular space, allowing paracrine activation of neighboring cells. In contrast, Type III NRG1 contains a cysteine-rich transmembrane domain that anchors the protein to the plasma membrane, where it primarily mediates juxtracrine signaling critical for axonal myelination and Schwann cell development [43,44,45]. Additional isoforms exhibit unique developmental expression patterns and specialized physiological roles, although their contributions to neuroinflammatory diseases remain less well characterized.

Increasing evidence suggests that different NRG1 isoforms are differentially regulated during injury and disease, raising the possibility that individual isoforms may exert distinct, and in some cases opposing, biological effects [7,9,35,36,37]. Importantly, nearly all mechanistic and therapeutic studies have relied on recombinant NRG1β1, leaving significant gaps in our understanding of the endogenous functions of alternative splice variants, α isoforms, membrane-bound isoforms, and intracellular signaling fragments. Defining the isoform-specific, receptor-selective, and cell-type-specific actions of NRG1 therefore represents a major priority for future translational research and the development of precision NRG1-based therapeutics.

### 2.2. NRG1 Proteolytic Processing and Ligand Availability

NRG1 signaling is tightly regulated by post-translational proteolytic processing. Most NRG1 isoforms are synthesized as membrane-associated precursor proteins that require extracellular cleavage before receptor activation can occur. This regulated proteolytic processing provides an additional level of temporal and spatial control over NRG1 signaling. The principal sheddases responsible for NRG1 activation include members of the A Disintegrin and Metalloprotease (ADAM) family, particularly ADAM17 (TACE) and ADAM19, as well as the β-site amyloid precursor protein cleaving enzyme BACE1 [9,46,47]. Cleavage by ADAM proteases releases soluble NRG1, which is capable of activating nearby ErbB receptors through paracrine signaling, whereas BACE1-mediated cleavage has been shown to regulate axon-glia communication and myelination within both the central and peripheral nervous systems.

Following ectodomain shedding, additional processing by γ-secretase may liberate intracellular fragments capable of participating in nuclear signaling and transcriptional regulation, suggesting that NRG1 signaling extends beyond classical receptor activation [43,48]. Dysregulation of these proteolytic pathways has been implicated in neurodevelopmental disorders, Alzheimer’s disease, schizophrenia, and demyelinating diseases, highlighting the importance of ligand processing as a determinant of biological activity [49]. As different proteases exhibit distinct tissue distributions and substrate specificities, alterations in proteolytic processing may substantially influence both the magnitude and the quality of NRG1 signaling during neuroinflammation.

### 2.3. ErbB Receptor Biology

The biological actions of NRG1 are mediated through members of the ErbB receptor tyrosine kinase family, consisting of four structurally related receptors: ErbB1 (EGFR), ErbB2 (HER2), ErbB3, and ErbB4. Among these receptors, NRG1 primarily binds ErbB3 and ErbB4, which subsequently form homo- or heterodimers capable of activating diverse intracellular signaling pathways [8,9,10,50,51]. Each receptor possesses unique structural and functional properties that contribute to signaling specificity. ErbB2 has no known endogenous ligand but functions as the preferred heterodimerization partner for other ErbB receptors, producing highly stable receptor complexes with prolonged signaling activity. Conversely, ErbB3 exhibits impaired intrinsic kinase activity and relies on heterodimerization with ErbB2 or ErbB4 to initiate downstream signaling. ErbB4 possesses both ligand-binding and kinase activity, enabling it to function independently or in combination with other family members.

The composition of receptor dimers profoundly influences downstream biological responses. ErbB4-containing receptor complexes are expressed in neurons, oligodendrocytes, and subsets of microglia and astrocytes, and in several experimental systems their activation has been associated with neuroprotective or pro-repair signaling [14,15,19,52,53,54,55,56,57]. By contrast, ErbB2-containing receptor complexes have been implicated in spinal microglial activation and neuropathic pain through MEK/ERK signaling and pro-inflammatory mediator production [30]. These observations support receptor composition as one determinant of NRG1 function, while also emphasizing that cell type, ligand isoform, disease state, and receptor abundance influence signaling output.

### 2.4. ErbB Receptor Downstream Signaling Pathways

Activation of ErbB receptors initiates an intricate network of intracellular signaling pathways that collectively regulate cell survival, metabolism, inflammation, proliferation, differentiation, and tissue repair. Rather than acting through a single signaling cascade, NRG1 simultaneously coordinates multiple interconnected pathways whose relative contributions vary according to cell type and physiological context. One of the best-characterized pathways involves activation of phosphatidylinositol 3-kinase (PI3K)/Akt, which promotes neuronal survival by inhibition of apoptotic signaling, preservation of mitochondrial function, and enhancement of cellular metabolism [11,12,22,58]. Activation of Akt also stimulates mammalian target of rapamycin (mTOR)-dependent protein synthesis while suppressing glycogen synthase kinase-3β (GSK-3β), thereby supporting axonal growth, synaptic plasticity, and remyelination.

Parallel activation of the mitogen-activated protein kinase (MAPK)/ERK pathway regulates cellular proliferation, differentiation, and survival. Although ERK signaling contributes to neuronal repair and oligodendrocyte maturation following CNS injury, excessive or prolonged ERK activation within spinal microglia has been implicated in chronic pain sensitization, illustrating the context-dependent nature of NRG1 signaling [56,59]. Additional signaling pathways include Janus kinase (JAK)/STAT, AMP-activated protein kinase (AMPK), cyclic AMP response element-binding protein (CREB), Forkhead box O (FOXO) transcription factors, and nuclear factor-kappa B (NF-κB) [13,14,20,60].

Among these, modulation of NF-κB signaling is one of the better-supported mechanisms associated with the anti-inflammatory effects reported for NRG1 in selected preclinical models. Experimental studies have shown reduced NF-kB activation and lower expression of pro-inflammatory mediators including tumor necrosis factor-α (TNF-α), interleukin (IL)-1β, IL-6, inducible nitric oxide synthase (iNOS), and cyclooxygenase-2 (COX-2) after NRG1 treatment in specific injury paradigms [12]. These findings should not be interpreted as evidence that NRG1 uniformly suppresses inflammatory signaling across all tissues or disease states.

Emerging evidence also implicates NRG1 in the regulation of mitochondrial homeostasis and autophagy through activation of AMPK and downstream autophagic pathways involving ULK1, ATG5, and LC3B. These mechanisms may contribute to the removal of damaged organelles, attenuation of oxidative stress, and restoration of cellular homeostasis following CNS injury [61].

Rather than functioning as isolated signaling modules, these pathways exhibit extensive cross-talk that enables NRG1 to coordinate complex multicellular responses during neuroinflammation. The integration of survival signaling, inflammatory regulation, metabolic adaptation, and regenerative programs positions NRG1 as a key regulator of neuroimmune homeostasis rather than a conventional neurotrophic factor.

### 2.5. Context-Dependent Signaling: A Unifying Framework

An emerging concept in NRG1 biology is that biological outcomes are determined not by NRG1 itself but by the cellular environment in which signaling occurs. Receptor expression patterns, ligand isoforms, developmental stage, disease severity, inflammatory milieu, and regional neuroanatomy collectively shape downstream responses. Within the injured brain, NRG1 predominantly activates ErbB4-dependent signaling that suppresses excessive inflammation, stabilizes the neurovascular unit, promotes oligodendrocyte differentiation, and facilitates neuronal survival [36,54,62,63,64,65,66,67]. These coordinated actions favor resolution of inflammation and tissue repair following ischemic stroke, traumatic brain injury, and chronic neurodegenerative disorders. In contrast, activation of ErbB2-containing receptor complexes within spinal nociceptive pathways enhances microglial activation, MEK/ERK signaling, and central sensitization, contributing to the development and maintenance of neuropathic pain.

**Figure 1:**
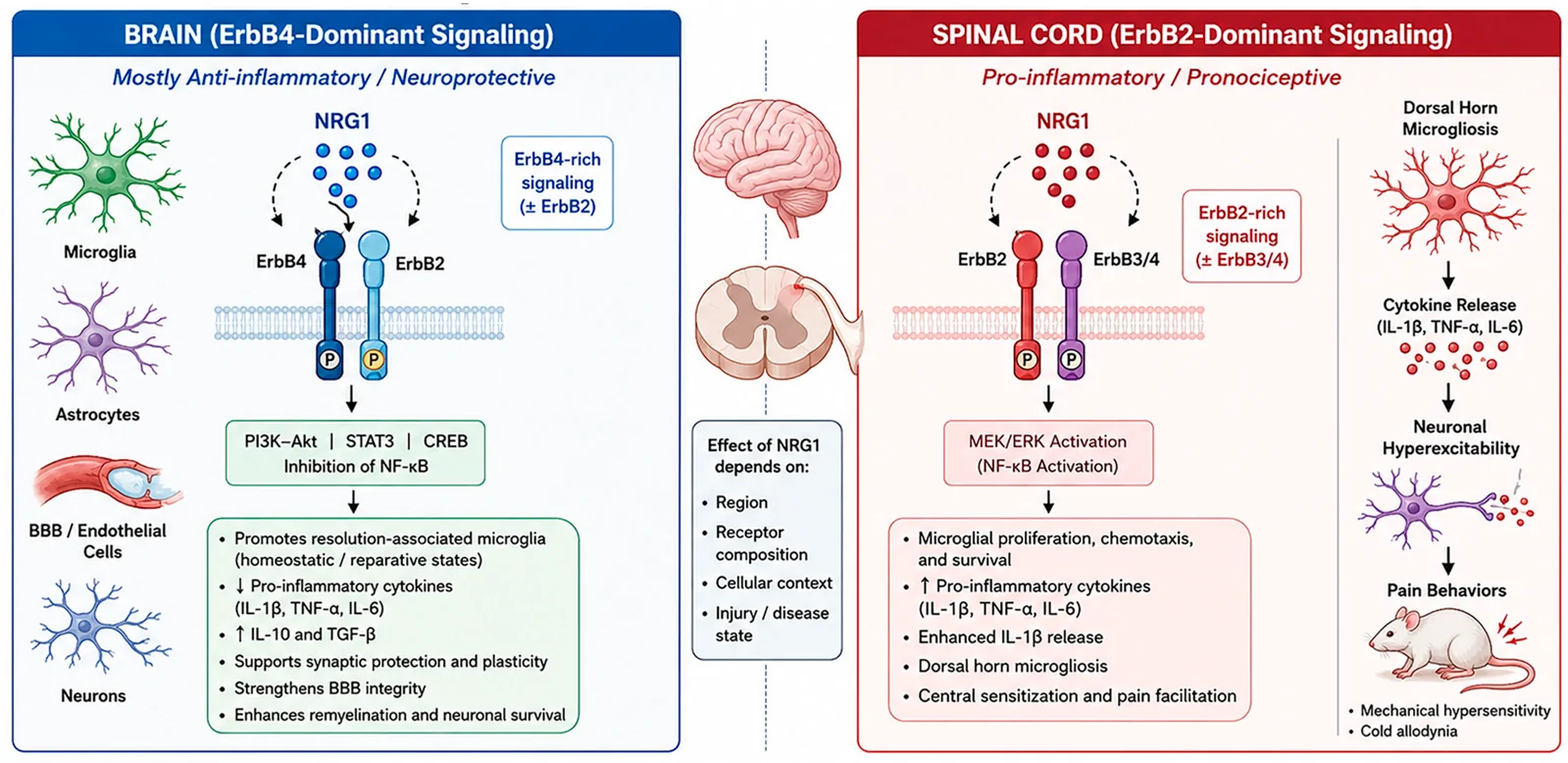
NRG1 signaling is highly context-dependent: Protective in the brain through ErbB4, but pronociceptive in the spinal cord through ErbB2.

This context-dependent model reconciles many of the seemingly contradictory findings reported in the literature and provides a conceptual framework for understanding the diverse biological actions of NRG1 across neurological diseases (Figure 1). Rather than representing fundamentally opposing effects, these divergent responses likely reflect differences in receptor composition, cellular targets, and regional signaling networks. Consequently, future therapeutic strategies should focus on selectively modulating receptor- and cell-specific signaling pathways to maximize neuroprotection while minimizing adverse outcomes.

## 3. Molecular Mechanisms Underlying the Immunomodulatory and Neuroprotective Actions of NRG-1

The biological actions of NRG1 are mediated through a highly integrated network of intracellular signaling pathways that regulate immune activation, cellular metabolism, oxidative stress, vascular integrity, and tissue regeneration [9,10]. Unlike conventional anti-inflammatory agents that inhibit a single cytokine or signaling pathway, NRG1 coordinates multiple cellular responses across neurons, glia, endothelial cells, and infiltrating immune cells to restore CNS homeostasis following injury. The pleiotropic nature of NRG1 signaling allows simultaneous attenuation of pathological inflammation while promoting endogenous repair processes, making it uniquely suited for treating complex neurological diseases characterized by multifactorial injury mechanisms.

Accumulating evidence indicates that these diverse biological effects arise from coordinated modulation of several interconnected signaling networks, including PI3K/Akt, MAPK/ERK, NF-κB, CREB, FOXO, AMPK, and α7 nicotinic acetylcholine receptor (α7nAChR)-dependent pathways [37,41,68]. Importantly, the relative contribution of each pathway varies according to cell type, receptor composition, disease stage, and tissue microenvironment, reinforcing the concept that NRG1 functions as a context-dependent regulator of neuroimmune homeostasis.

### 3.1. Regulation of PI3K/Akt Signaling

Activation of the PI3K/Akt pathway represents one of the earliest and most consistently observed molecular responses following NRG1 stimulation. Binding of NRG1 to ErbB receptor complexes induces receptor autophosphorylation, creating docking sites for PI3K, which subsequently generates phosphatidylinositol (3,4,5)-trisphosphate (PIP3) and recruits Akt to the plasma membrane for activation [15,16,19,56].

Akt functions as a master regulator of cellular survival by phosphorylating numerous downstream substrates involved in apoptosis, metabolism, protein synthesis, and inflammatory signaling. NRG1-mediated Akt activation inhibits pro-apoptotic proteins including Bad, caspase-9, and GSK-3β, while simultaneously enhancing expression of anti-apoptotic proteins such as Bcl-2 and Bcl-xL. These effects preserve mitochondrial integrity, reduce cytochrome c release, and prevent activation of intrinsic apoptotic pathways following ischemic and traumatic CNS injury.

Beyond promoting neuronal survival, PI3K/Akt signaling contributes to oligodendrocyte differentiation, remyelination, axonal regeneration, and endothelial cell survival [15,19]. Activation of Akt also stimulates mammalian target of rapamycin (mTOR)-dependent protein synthesis while coordinating metabolic adaptations required for tissue repair. Experimental studies consistently demonstrate that pharmacological inhibition of PI3K significantly attenuates the neuroprotective effects of NRG1, highlighting the central role of this pathway in mediating functional recovery [16,17,55,59,69,70].

### 3.2. MAPK/ERK Signaling: Repair Versus Pathological Activation

MAPK/ERK signaling represents another major downstream target of ErbB receptor activation. The biological consequences of ERK activation, however, are highly context-dependent and illustrate the dual nature of NRG1 signaling. Within neurons and oligodendrocytes, transient activation of ERK promotes cellular proliferation, differentiation, neurite extension, synaptic plasticity, and myelin formation. ERK signaling also contributes to angiogenesis and vascular remodeling following ischemic injury, facilitating restoration of tissue perfusion and repair [56,59,71].

Conversely, persistent ERK activation within spinal microglia has been implicated in chronic neuropathic pain. Following peripheral nerve injury, NRG1 released from damaged primary afferent neurons activates ErbB2-containing receptor complexes on spinal microglia, leading to sustained MEK/ERK activation, increased production of TNF-α, IL-1β, brain-derived neurotrophic factor (BDNF), and enhanced central sensitization [30,43,72,73,74]. These observations demonstrate that the biological consequences of ERK activation are determined by receptor composition, cell type, and anatomical location rather than by ERK signaling itself.

### 3.3. Inhibition of NF-κB Signaling

Suppression of NF-κB signaling is one of the principal mechanisms proposed to underlie the anti-inflammatory effects of NRG1 in several preclinical models [12,24,30,75,76]. NF-κB serves as a major transcriptional regulator of inflammatory mediators, including TNF-α, IL-1β, IL-6, iNOS, COX-2, chemokines, and adhesion molecules. Studies in ischemic stroke, traumatic brain injury, cerebral malaria, and other cell-based inflammatory models have reported that NRG1 reduces phosphorylation or nuclear translocation of NF-κB p65 and preserves inhibitor of κB (IκB) expression after NRG1 treatment [12,14,24]. Consequently, transcription of multiple pro-inflammatory genes is suppressed, resulting in decreased cytokine production, reduced leukocyte recruitment, diminished oxidative stress, and attenuation of secondary tissue injury. However, these observations are model-dependent and do not establish universal NF-κB inhibition by NRG1.

Importantly, inhibition of NF-κB by NRG1 does not appear to completely abolish inflammatory signaling. Rather, NRG1 selectively limits excessive inflammatory amplification while preserving immune functions required for debris clearance and tissue remodeling. This balanced regulation likely contributes to the superior therapeutic profile of NRG1 compared with broad-spectrum immunosuppressive agents.

### 3.4. CREB and FOXO Transcriptional Programs

Recent transcriptomic analyses have identified CREB and FOXO transcription factors as important downstream mediators of NRG1 signaling. Activation of CREB promotes transcription of genes involved in neuronal survival, synaptic plasticity, mitochondrial function, and anti-inflammatory responses, whereas FOXO proteins regulate oxidative stress resistance, autophagy, DNA repair, and cellular metabolism. Our previous genomic analyses following experimental ischemic stroke demonstrated that NRG1 treatment increased CREB1 and FOXO1 transcriptional activity while simultaneously suppressing inflammatory transcriptional networks regulated by NF-κB [20]. These coordinated transcriptional changes suggest that NRG1 actively reprograms injured tissues toward a pro-survival, anti-inflammatory phenotype rather than simply inhibiting inflammatory signaling. The convergence of CREB, FOXO, and Akt signaling provides a mechanistic explanation for the broad spectrum of neuroprotective effects observed across multiple experimental models.

### 3.5. AMPK, Autophagy, and Mitochondrial Homeostasis

Mitochondrial dysfunction and impaired energy metabolism are fundamental drivers of neurodegeneration following ischemic and inflammatory injury. Emerging evidence indicates that NRG1 promotes mitochondrial quality control through activation of AMPK and downstream autophagic pathways [61]. AMPK functions as a cellular energy sensor that coordinates metabolic adaptation during stress. Activation of AMPK stimulates autophagy through phosphorylation of ULK1 while suppressing mTOR activity under conditions of energy depletion. Subsequent activation of ATG5, LC3B, and related autophagic proteins facilitates removal of damaged mitochondria and aggregated proteins, reducing oxidative stress and restoring cellular homeostasis. These mechanisms are particularly relevant in ischemic stroke, Parkinson’s disease, Alzheimer’s disease, and traumatic brain injury, where mitochondrial dysfunction contributes substantially to progressive neuronal degeneration.

### 3.6. Modulation of the Cholinergic Anti-Inflammatory Pathway

An emerging mechanism underlying the anti-inflammatory actions of NRG1 involves activation of the cholinergic anti-inflammatory pathway through regulation of the α7nAChR. Activation of α7nAChR suppresses cytokine production by macrophages and microglia through inhibition of NF-κB signaling and reduced activation of inflammasome pathways [77,78,79,80,81]. Experimental studies indicate that NRG1 enhances α7nAChR expression and signaling, thereby amplifying endogenous mechanisms of inflammatory resolution [23]. Because cholinergic signaling simultaneously regulates inflammation, synaptic transmission, and cognitive function, interactions between NRG1 and α7nAChR may contribute to both neuroprotection and functional recovery following CNS injury. Additional investigation is warranted to determine whether receptor-specific modulation of this pathway may provide synergistic therapeutic benefits.

### 3.7. Oxidative Stress and Redox Homeostasis

Reactive oxygen species (ROS) generated during ischemia and chronic neuroinflammation contribute to lipid peroxidation, DNA damage, mitochondrial dysfunction, and activation of inflammatory signaling cascades. NRG1 has been shown to reduce oxidative stress through multiple complementary mechanisms [21,59,69,75,82]. Activation of PI3K/Akt and CREB signaling enhances expression of antioxidant enzymes, including superoxide dismutase, catalase, glutathione peroxidase, and heme oxygenase-1 [59]. Simultaneously, suppression of NF-κB signaling reduces inflammatory ROS production by activated microglia and infiltrating leukocytes [21,75]. Through these coordinated actions, NRG1 limits oxidative injury while preserving mitochondrial function and promoting long-term neuronal survival.

### 3.8. Integrated Signaling Network

**Figure 2:**
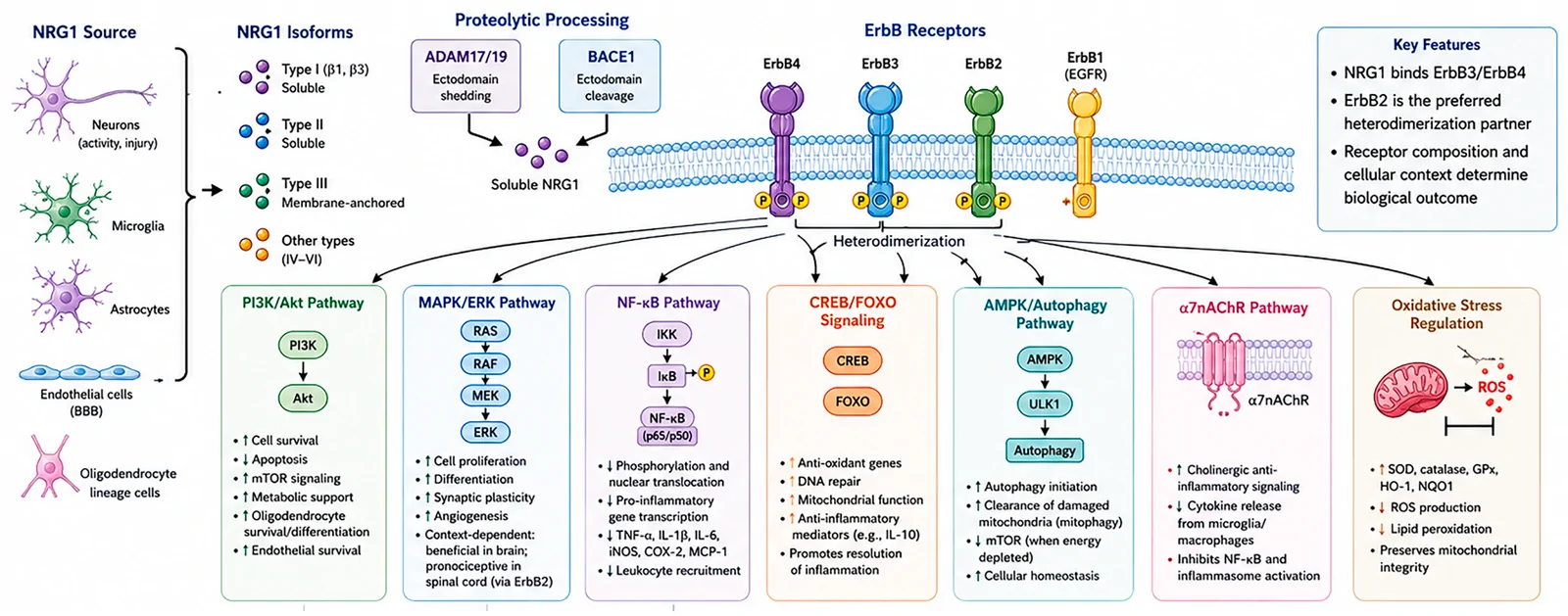
Integrated molecular mechanisms of NRG1 signaling in neuroinflammation. This systems-level coordination distinguishes NRG1 from conventional anti-inflammatory therapies that target single cytokines or receptors. Instead of simply suppressing inflammation, NRG1 appears to orchestrate the transition from acute inflammatory injury to tissue repair by synchronizing protective responses across multiple CNS cell populations. Consequently, therapeutic strategies that selectively engage receptor- and cell-specific NRG1 signaling pathways may achieve superior neuroprotection while minimizing adverse effects associated with global immune suppression.

Although individual signaling pathways are often studied independently, NRG1 should be viewed as coordinating an integrated molecular network rather than activating isolated signaling cascades. Crosstalk among PI3K/Akt, MAPK/ERK, NF-κB, CREB, FOXO, AMPK, and α7nAChR signaling enables simultaneous regulation of inflammation, metabolism, vascular function, myelination, synaptic plasticity, and tissue repair (Figure 2).

## 4. Cell-Specific Effects of Neuregulin-1 in Neuroinflammation and Neural Repair

The therapeutic effects of NRG1 arise not from actions on a single cellular target but from its ability to coordinate signaling among multiple components of the neurovascular unit. Neurons, microglia, astrocytes, oligodendrocytes, endothelial cells, and infiltrating peripheral immune cells all express varying combinations of ErbB receptors and respond differently to NRG1 depending on receptor composition, developmental stage, and the inflammatory microenvironment. This multicellular response distinguishes NRG1 from conventional anti-inflammatory therapies that primarily target individual cytokines or immune pathways.

An emerging concept is that NRG1 can function as a systems-level regulator of neuroimmune homeostasis by influencing communication among resident CNS and vascular cells. Across different experimental settings, NRG1 has been associated with reduced inflammatory signaling together with effects on BBB integrity, remyelination, synaptic plasticity, and functional recovery. Whether these actions represent coordinated multicellular reprogramming in vivo remains incompletely established and likely varies by disease context.

### 4.1. Microglia: Central Regulators of Neuroimmune Homeostasis

Microglia are the principal innate immune cells of the CNS and represent one of the primary cellular targets of NRG1 [2,32,83,84,85]. Under physiological conditions, homeostatic microglia continuously survey the CNS microenvironment, remove apoptotic cells, remodel synapses, and maintain tissue homeostasis. Following injury or disease, however, microglia rapidly transition into activated states characterized by increased production of inflammatory cytokines, chemokines, ROS, and proteolytic enzymes.

Although historically categorized into pro-inflammatory (M1) and anti-inflammatory (M2) phenotypes, recent single-cell transcriptomic studies have demonstrated that microglial activation exists along a dynamic continuum that includes disease-associated microglia (DAM), interferon-responsive microglia, proliferative-region-associated microglia, and multiple intermediate activation states [86,87]. This evolving understanding emphasizes that therapeutic strategies should promote appropriate inflammatory resolution rather than broadly suppress microglial activation.

Accumulating evidence indicates that NRG1 can alter microglial inflammatory phenotypes in selected experimental models, including through suppression of NF-κB signaling and activation of PI3K/Akt-associated pathways [12,24,30,75,76]. Reported effects include reductions in TNF-α, IL-1β, IL-6, iNOS, and COX-2 and changes in oxidative and inflammatory signaling. Because contemporary single-cell studies show that microglial states extend beyond a simple M1/M2 framework, these findings are more appropriately interpreted as context-dependent shifts in activation programs rather than definitive ‘reprogramming’ toward a single reparative phenotype.

Importantly, the effects of NRG1 on microglia appear to be receptor-dependent. In the injured brain, signaling through ErbB4-containing receptor complexes generally suppresses inflammatory activation and promotes tissue repair [14,18,24,76,88,89,90]. In contrast, following peripheral nerve injury, NRG1 activates ErbB2-containing receptor complexes on spinal microglia, stimulating MEK/ERK signaling and contributing to neuropathic pain [30,72,73,74]. This receptor-specific divergence highlights the context-dependent nature of NRG1 signaling and underscores the need for receptor-selective therapeutic strategies.

### 4.2. Astrocytes: Coordinators of Neurovascular and Metabolic Homeostasis

Astrocytes perform essential roles in maintaining CNS homeostasis through regulation of neurotransmitter recycling, metabolic support, ion buffering, antioxidant defense, and BBB integrity. Following injury, astrocytes undergo reactive transformation characterized by hypertrophy, altered gene expression, and production of inflammatory mediators. While reactive astrocytes initially limit tissue damage, persistent activation contributes to chronic inflammation, glial scar formation, and impaired neuronal regeneration.

Neuregulin has several reported effects on astrocytes, and these effects are context-dependent. In injury settings, reactive astrocytes can express or respond to neuregulin signaling, which may support astrocyte proliferation and neuronal survival around lesions [13,67,91,92]. In spinal cord injury models, NRG1 has been reported to push reactive astrocytes toward an oligodendrocyte-lineage-like state through PI3K-AKT-mTOR signaling, suggesting a role in repair and remyelination rather than only inflammation control [93]. Astrocytes may also contribute to the neurovascular and trophic actions of NRG1, since NRG1-ErbB signaling has been linked to protection of astrocytes from injury and to broader support of BBB integrity and glial-neuronal communication.

### 4.3. Oligodendrocytes and Oligodendrocyte Precursor Cells

Among CNS cell populations, oligodendrocytes exhibit particularly strong dependence on NRG1 signaling for survival, maturation, and myelin maintenance. Oligodendrocytes provide metabolic support to axons while generating myelin sheaths that facilitate rapid saltatory conduction. Loss of oligodendrocytes contributes to functional deficits in multiple sclerosis, ischemic stroke, traumatic brain injury, and numerous neurodegenerative disorders.

NRG1 promotes proliferation and differentiation of oligodendrocyte precursor cells (OPCs) through activation of PI3K/Akt and ERK signaling pathways. These effects increase expression of myelin proteins including myelin basic protein (MBP), proteolipid protein (PLP), and myelin-associated glycoprotein (MAG), thereby accelerating remyelination following injury [43,44,54]. In addition to stimulating myelin formation, NRG1 enhances oligodendrocyte survival by suppressing apoptosis, reducing oxidative stress, and improving mitochondrial function [94,95,96]. These actions preserve white matter integrity while maintaining axonal metabolic support. Because remyelination represents one of the few endogenous repair processes capable of restoring neurological function after injury, the effects of NRG1 on oligodendrocyte biology likely contribute substantially to its long-term therapeutic benefits.

### 4.4. Neurons: Neuroprotection and Synaptic Plasticity

Although the immunomodulatory effects of NRG1 have received increasing attention, neurons remain important direct targets of NRG1 signaling. Neuronal ErbB4 receptors regulate neuronal survival, dendritic development, synaptic maturation, neurotransmitter release, and circuit plasticity throughout development and adulthood [8,97,98,99].

Following CNS injury, activation of PI3K/Akt and CREB signaling by NRG1 promotes neuronal survival through inhibition of apoptotic pathways, preservation of mitochondrial integrity, and enhancement of antioxidant defenses. NRG1 also stimulates axonal regeneration, dendritic remodeling, and synaptogenesis, thereby facilitating recovery of functional neural networks after ischemic and traumatic injury [14,16,17,19,55,59,69,100].

NRG1 additionally modulates excitatory and inhibitory neurotransmission through regulation of glutamatergic and GABAergic synapses. These effects may contribute to restoration of circuit homeostasis while limiting excitotoxic injury during acute neuroinflammation [52,53,89,101,102,103].

### 4.5. Endothelial Cells and the Neurovascular Unit

The BBB is increasingly recognized as an active participant in neuroinflammatory diseases rather than a passive structural barrier. Endothelial cells, pericytes, astrocytic endfeet, basement membrane components, and resident immune cells collectively form the neurovascular unit, which regulates immune cell trafficking, nutrient transport, and maintenance of CNS homeostasis [104].

NRG1 exerts profound protective effects on the neurovascular unit [22,105,106,107]. Activation of ErbB signaling preserves endothelial cell survival, enhances expression of tight junction proteins including claudin-5, occludin, and zonula occludens-1 (ZO-1), and reduces endothelial permeability following ischemic injury. NRG1 also suppresses expression of vascular adhesion molecules that promote leukocyte infiltration into the CNS. These vascular effects are particularly important because BBB disruption often precedes widespread neuroinflammation. By maintaining vascular integrity, NRG1 limits secondary inflammatory amplification while preserving cerebral perfusion and reducing vasogenic edema.

### 4.6. Cell–Cell Crosstalk: An Integrated Neuroimmune Network

**Figure 3:**
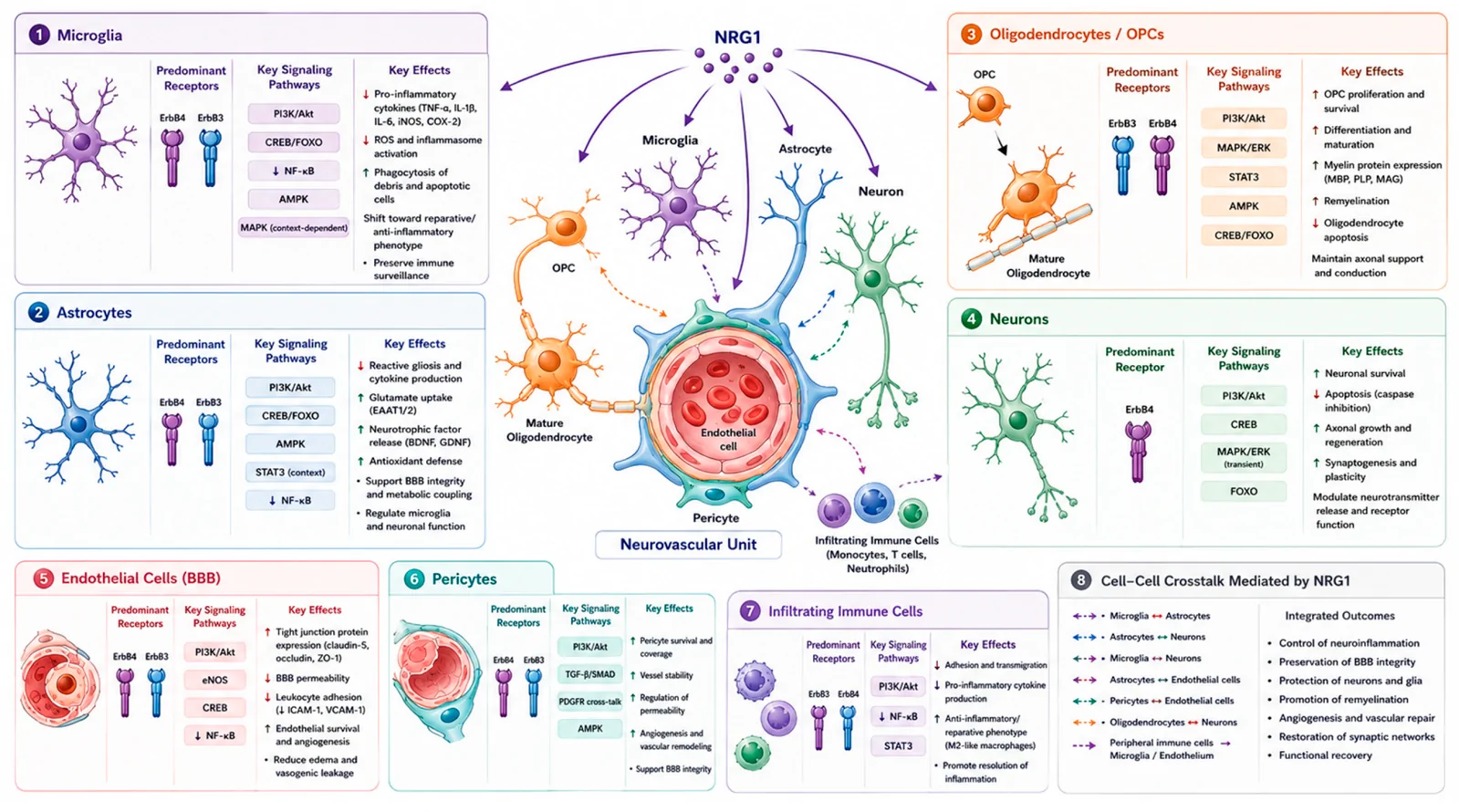
Cell-specific actions of NRG-1 within the neurovascular unit.

A major conceptual advance emerging from recent studies is that NRG1 should not be viewed as acting independently on individual cell types. Instead, NRG1 coordinates extensive communication among neurons, glia, vascular cells, and infiltrating immune cells to restore tissue homeostasis (Figure 3). For example, NRG1-mediated suppression of microglial cytokine production reduces astrocyte activation, which subsequently enhances BBB integrity and decreases leukocyte infiltration. Improved vascular stability limits inflammatory amplification while promoting oligodendrocyte survival and neuronal repair. Simultaneously, neuronal release of trophic factors further reinforces glial repair programs, creating positive feedback loops that facilitate recovery.

Taken together, these observations suggest that NRG1 may influence communication among neural, glial, and vascular compartments rather than acting on a single cellular target. However, the causal sequence and relative contribution of these cell–cell interactions remain incompletely resolved and should be tested directly in disease-relevant models.

## 5. Disease Applications of Neuregulin-1: A Context-Dependent Neuroimmune Therapeutic

The diverse biological actions of NRG1 are reflected in its therapeutic potential across a broad spectrum of neurological disorders characterized by neuroinflammation, neurovascular dysfunction, demyelination, and progressive neuronal loss. Although these diseases differ considerably in their initiating insults and pathological features, they share common mechanisms of secondary injury, including activation of resident glial cells, disruption of BBB integrity, oxidative stress, mitochondrial dysfunction, and chronic inflammatory signaling. Rather than targeting disease-specific pathogenic proteins, NRG1 appears to modulate conserved cellular programs that regulate inflammatory resolution and tissue repair. Consequently, the therapeutic effects of NRG1 are best understood within a framework of shared neuroimmune mechanisms rather than as isolated disease-specific observations.

Importantly, the strength of evidence supporting NRG1 therapy varies considerably among neurological disorders. Robust data exist for ischemic stroke and experimental demyelinating disease, whereas studies in AD, PD, cerebral malaria, and sickle cell disease remain comparatively limited. Furthermore, the mechanisms responsible for therapeutic benefit are not identical across diseases, emphasizing that NRG1 signaling is highly dependent on cellular context, receptor composition, and disease stage.

### 5.1. Ischemic Stroke

Among neurological disorders, ischemic stroke represents the most extensively investigated application of NRG1 therapy. Stroke initiates a complex cascade of excitotoxicity, oxidative stress, mitochondrial dysfunction, BBB disruption, leukocyte infiltration, and sustained neuroinflammation that contributes substantially to secondary neuronal injury [9,61,108]. Numerous experimental studies have demonstrated that NRG1 attenuates multiple components of this injury cascade, resulting in reduced infarct volume, improved neurological recovery, and enhanced long-term tissue preservation [9,10,34,109,110].

Mechanistically, NRG1 suppresses activation of NF-κB-dependent inflammatory pathways while simultaneously activating PI3K/Akt, CREB, and FOXO signaling, thereby promoting neuronal survival and limiting apoptosis [12,20,57,109,111]. NRG1 also reduces microglial activation, decreases expression of TNF-α, IL-1β, IL-6, and iNOS, preserves tight junction proteins within the BBB, and reduces infiltration of peripheral inflammatory cells. These coordinated effects stabilize the neurovascular unit and limit expansion of secondary injury.

Beyond acute neuroprotection, experimental studies have associated NRG1 treatment with oligodendrocyte survival, remyelination, angiogenesis, axonal remodeling, and synaptic plasticity [10,28,29,61]. Transcriptomic and proteomic studies further suggest that NRG1 can shift injured tissue toward gene-expression programs associated with inflammatory resolution and repair, although the extent to which this represents durable cellular reprogramming remains to be established.

One of the most clinically significant observations is the relatively prolonged therapeutic window reported for recombinant NRG1 in experimental stroke models. Unlike tissue plasminogen activator (tPA), which must be administered within a narrow time window, NRG1 has demonstrated efficacy when administered several hours after ischemic onset in multiple preclinical studies [10,34,110]. These findings support continued investigation of NRG1 as an adjunctive neuroprotective therapy alongside reperfusion strategies such as thrombolysis and mechanical thrombectomy.

### 5.2. Traumatic Brain Injury

Traumatic brain injury (TBI) is characterized by an immediate mechanical insult followed by prolonged secondary injury involving neuroinflammation, oxidative stress, diffuse axonal injury, BBB disruption, and progressive neurodegeneration. Because secondary injury evolves over days to weeks, therapeutic strategies targeting inflammatory resolution remain attractive.

Experimental studies demonstrate that NRG1 reduces activation of microglia and astrocytes, suppresses inflammatory cytokine production, preserves BBB integrity, and decreases neuronal apoptosis following experimental TBI [27,89,112,113]. Activation of PI3K/Akt and CREB signaling promotes neuronal survival while limiting oxidative injury and mitochondrial dysfunction. Additional studies suggest that NRG1 enhances synaptic remodeling and axonal regeneration during later phases of recovery. Although available data remain more limited than in stroke, existing evidence suggests that NRG1 may influence both acute neuroprotection and chronic repair processes following traumatic injury.

### 5.3. Multiple Sclerosis and Demyelinating Disease

Multiple sclerosis represents one of the strongest biological rationales for NRG1 therapy because the molecule regulates both immune responses and oligodendrocyte biology. Demyelination results from inflammatory destruction of oligodendrocytes followed by incomplete remyelination and chronic axonal degeneration.

NRG1 promotes proliferation and differentiation of OPCs, enhances expression of myelin proteins including MBP and PLP, and accelerates remyelination following experimental demyelination [41,51,114,115]. Simultaneously, NRG1 reduces microglial activation, suppresses inflammatory cytokine production, and limits leukocyte infiltration into the CNS.

Experimental autoimmune encephalomyelitis (EAE) studies demonstrate improved neurological function accompanied by reduced inflammatory lesions and enhanced white matter repair [115]. Because remyelination remains an unmet therapeutic goal in progressive MS, NRG1-mediated activation of endogenous repair pathways represents an attractive strategy that complements current immunomodulatory therapies.

### 5.4. Alzheimer’s Disease

Neuroinflammation is increasingly recognized as a central contributor to Alzheimer’s disease progression. Activated microglia, reactive astrocytes, chronic cytokine production, BBB dysfunction, and impaired synaptic plasticity all contribute to progressive cognitive decline [116].

Experimental studies indicate that NRG1 reduces microglial activation, attenuates oxidative stress, improves mitochondrial function, enhances synaptic plasticity, and protects hippocampal neurons from amyloid-β-mediated toxicity. NRG1 also appears to regulate neuronal connectivity through effects on dendritic spine maintenance and neurotransmission [18,117]. Human pathological studies show ErbB4 expression in plaque-associated microglia and reactive astrocytes, indicating that endogenous NRG1-ErbB signaling may be engaged in diseased tissue [117,118,119]. However, these observations do not establish therapeutic efficacy in humans.

Evidence that NRG1 directly modifies amyloid or tau pathology remains inconsistent. Current data are largely preclinical and more strongly support effects on downstream inflammatory, synaptic, and neurodegenerative processes than on the initiating proteinopathies themselves. Accordingly, the disease-modifying potential of NRG1 in Alzheimer’s disease remains uncertain.

### 5.5. Parkinson’s Disease

Parkinson’s disease involves progressive degeneration of dopaminergic neurons accompanied by chronic activation of microglia and sustained production of inflammatory mediators within the substantia nigra [116,120]. In cell and animal models, NRG1 has protected dopaminergic neurons through pathways involving PI3K/Akt signaling, oxidative stress, mitochondrial function, and inflammatory mediators [121,122,123,124]. Additional studies suggest that NRG1 enhances neuronal survival through interactions with astrocytes and modulation of glutamatergic signaling. The evidence remains predominantly preclinical, and relatively few studies incorporate α-synuclein pathology, limiting conclusions regarding disease-modifying potential.

### 5.6. Cerebral Malaria

Cerebral malaria is characterized by severe neuroinflammation, endothelial activation, BBB dysfunction, leukocyte sequestration, and neuronal injury despite parasite-directed treatment. In experimental cerebral malaria, recombinant NRG1 has improved survival while reducing inflammatory cytokine production and preserving BBB integrity without directly affecting parasitemia [14,107]. These data support modulation of host inflammatory and neurovascular responses as a plausible mechanism. However, the evidence derives from a limited number of pre-clinical studies, and no clinical efficacy or CNS safety data currently establish NRG1 as a therapy for human cerebral malaria.

### 5.7. Sickle Cell Disease

Neurological complications of sickle cell disease arise from chronic hemolysis, endothelial dysfunction, ischemia–reperfusion injury, oxidative stress, and systemic inflammation. These processes contribute to stroke, silent cerebral infarction, and progressive cognitive impairment. Recent studies have associated NRG1 with reduced inflammatory cytokine production, improved endothelial function, reduced oxidative stress, and protection against multi-organ injury in experimental sickle cell disease [125,126,127]. CNS-specific evidence is sparse and human therapeutic studies are lacking. Thus, NRG1 should presently be viewed as a mechanistically interesting candidate for further investigation rather than an established strategy for preventing neurological complications of sickle cell disease.

### 5.8. Neuropathic Pain: An Important Biological Exception

Neuropathic pain represents an important exception to the predominantly neuroprotective actions of NRG1 within the CNS. Following peripheral nerve injury, membrane-associated NRG1 released from damaged sensory neurons activates ErbB2-containing receptor complexes on spinal microglia, stimulating MEK/ERK signaling, inflammatory cytokine production, and central sensitization [30,43,72,73].

These observations demonstrate that NRG1 signaling is not universally anti-inflammatory. Outcomes vary with receptor composition, cell type, anatomical location, ligand form, and injury context. ErbB4-associated signaling has been linked to protective responses in several brain-injury models, whereas ErbB2-dependent signaling in spinal nociceptive pathways contributes to pain hypersensitivity. This divergence provides a rationale for investigating receptor-biased or tissue-targeted strategies, but the safety and efficacy of ErbB4-selective agonism remain hypotheses that require direct experimental and clinical validation.

### 5.9. Schizophrenia and Therapeutic Modulation of NRG1-ErbB Signaling

NRG1-ErbB4 signaling has long been implicated in schizophrenia through genetic association studies, post-mortem observations, and experimental models that link the pathway to GABAergic and glutamatergic neurotransmission, synaptic plasticity, and cortical circuit function [7,128,129]. Genetic and postmortem studies indicate that NRG1–ErbB4 signaling is disturbed in schizophrenia. In patients, ErbB4 levels are increased in the dorsolateral prefrontal cortex, a change usually regarded as a compensatory response to reduced NRG1 availability or altered isoform processing [7,68]. Mice with ErbB4 conditionally deleted in GABAergic interneurons display hyperactivity, have impaired sensorimotor gating, and show deficits in working memory and social behavior. The behavioral pattern observed is partly similar to that seen in schizophrenia-like symptoms and thus supports the view that diminished ErbB4 signaling in interneurons contributes to the development of the disorder [7,68,128,129].

The role of NRG1 in neuroinflammation in schizophrenia is not very well understood. In some patients, microglial activation and elevated cytokine levels have been observed. NRG1-driven anti-inflammatory signaling may help contain the low-grade neuroinflammation that contributes to abnormalities in synaptic pruning. Therapeutic strategies that focus on ErbB4 in GABAergic interneurons, for example those involving positive allosteric modulators of ErbB4 and isoform-selective NRG1 analogs, are still in early preclinical development and must address the challenge of crossing the CNS barrier while maintaining receptor selectivity. As with other uses of NRG1, ErbB4 signaling in neural circuits is highly context-dependent. Therefore, administering drugs to enhance this signaling pathway could inadvertently disrupt the delicate balance of inhibition necessary for normal circuit function. Nevertheless, the NRG1-ErbB4-GABAergic pathway is one of the most extensively validated genetic approaches in this therapeutic area [7,68,128,129].

Drug-development efforts have therefore included repurposing strategies designed to reduce NRG1-ErbB4 signaling. A multicenter randomized proof-of-concept trial evaluated adjunctive spironolactone, proposed to antagonize NRG1-ERBB4 signaling, in 90 patients with schizophrenia [130,131]. The prespecified primary analysis did not demonstrate significant superiority over placebo for working-memory improvement, although exploratory post hoc analyses suggested a possible signal and treatment was generally well tolerated. These results do not establish efficacy but provide clinical proof that the NRG1-ErbB axis is being investigated as a pharmacological target in psychiatry. Future work should define molecularly selected patient subgroups, directionality of pathway dysregulation, CNS target engagement, and whether receptor- or circuit-specific modulation can improve cognition without disrupting the physiological roles of NRG1 in neural development and synaptic function.

### 5.10. Comparative Perspective Across Neurological Diseases

**Figure 4:**
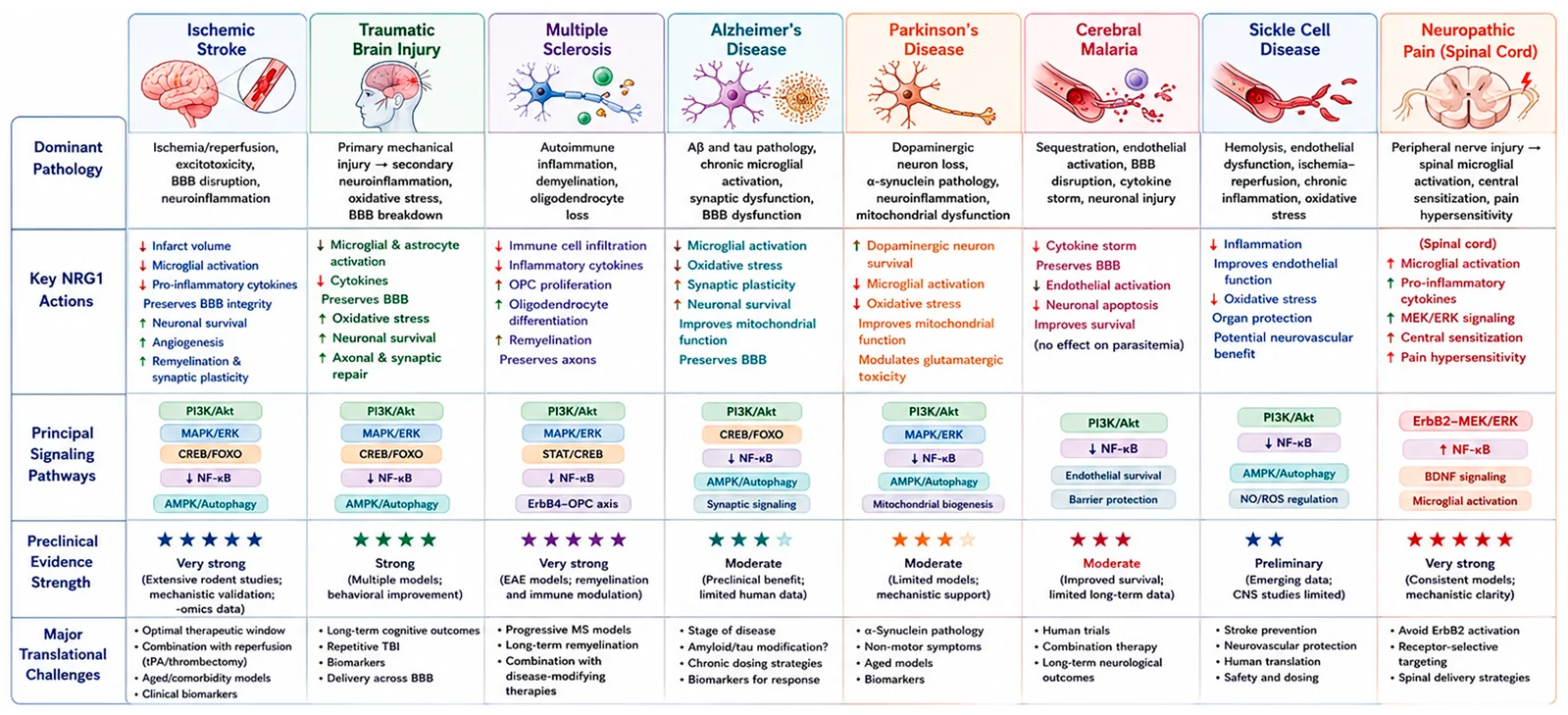
Disease-specific mechanisms and therapeutic applications of NRG1.

Across neurological disorders, the most reproducible preclinical themes are modulation of inflammatory signaling, preservation of neurovascular integrity, and support of neural or glial survival, but the relative contribution of each mechanism and the strength of evidence differ markedly by indication (Figure 4). Evidence is strongest in ischemic stroke and experimental demyelinating disease; TBI has a smaller but supportive pre-clinical literature, whereas Alzheimer’s disease, Parkinson’s disease, cerebral malaria, and sickle cell disease remain comparatively limited. Schizophrenia further illustrates that NRG1-ErbB signaling may need to be decreased rather than increased in some contexts. Thus, NRG1 is best viewed as a context-sensitive signaling axis whose therapeutic direction must be established for each disease rather than as a broadly applicable anti-inflammatory treatment.

This comparative perspective also highlights important translational considerations. The strongest evidence currently supports applications in ischemic stroke and demyelinating disease, whereas additional mechanistic and translational studies are needed in Alzheimer’s disease, Parkinson’s disease, cerebral malaria, and sickle cell disease. Across all conditions, future clinical success will likely depend on receptor-selective targeting, optimization of therapeutic timing, identification of predictive biomarkers, and integration of NRG1 with existing standard-of-care therapies.

## 6. Therapeutic Strategies and Translational Challenges

The preclinical literature positions NRG1 as a promising therapeutic platform for neurological disorders characterized by neuroinflammation, neurovascular dysfunction, demyelination, and neuronal loss. Its principal advantage is mechanistic breadth: NRG1 can simultaneously influence inflammatory signaling, endothelial integrity, oligodendrocyte survival, mitochondrial function, synaptic plasticity, and tissue repair. However, this same biological complexity creates substantial translational challenges. NRG1 is not a single, uniformly acting drug target but a family of structurally diverse ligands that signal through different ErbB receptor combinations across multiple organs and cell types. Therapeutic success will therefore depend on selectively engaging beneficial receptor-, cell-, and region-specific pathways while minimizing off-target signaling and maladaptive responses.

The most important translational question is not simply whether NRG1 signaling should be increased, but which NRG1 isoform, receptor complex, cellular target, dose, route, and therapeutic window should be selected for a particular disease context. This distinction is especially important given the predominantly protective effects associated with ErbB4-containing receptor complexes in the brain and the pronociceptive effects linked to ErbB2-dependent signaling in spinal microglia. Accordingly, future development should move beyond nonspecific enhancement of the NRG1–ErbB axis toward precision strategies designed to reproduce the beneficial components of NRG1 signaling.

### 6.1. Recombinant NRG1 in Clinical Trials and Delivery Considerations

Administration of recombinant NRG1 protein is the most direct therapeutic approach and has produced neuroprotective effects in several preclinical models. Human experience with recombinant NRG1 is derived largely from cardiovascular studies, including chronic heart failure and healthy-volunteer pharmacology, where recombinant NRG1-β1 or cimaglermin alfa was reported to be generally tolerated at studied doses [132,133,134,135,136]. These data do not establish neurological safety, CNS target engagement, or efficacy. No NRG1-based therapy is currently established for a neurological indication. CNS translation faces additional obstacles, including BBB penetration, receptor distribution, dose-dependent peripheral effects, and the possibility of engaging different ErbB receptor complexes outside the intended tissue. NRG1-beta can cross the BBB under some experimental conditions, and BBB disruption may alter exposure [122,124,137,138], but therapeutic brain concentrations after systemic dosing in humans remain unestablished. Neurological development will therefore require dedicated CNS pharmacokinetic, pharmacodynamic, safety, and proof-of-mechanism studies rather than extrapolation from cardiovascular trials.

### 6.2. Receptor-Selective Agonists and Biased Signaling

Receptor selectivity is a plausible strategy for improving the therapeutic index of NRG1-based interventions, but it remains an experimental objective rather than an established solution. Although NRG1 binds directly to ErbB3 and ErbB4, biological responses are strongly influenced by receptor dimerization, receptor abundance, and cellular context.

In neurological injury, selectively favoring ErbB4-associated signaling could, in principle, preserve some neuroprotective and reparative effects reported in brain models while reducing unwanted ErbB2-dependent signaling. Candidate approaches include engineered ligands, receptor-targeted antibodies, bivalent molecules, and pathway-biased strategies. At present, however, there is insufficient evidence to conclude that any ErbB4-selective approach will reproduce the beneficial actions of NRG1 without introducing new liabilities; these concepts require direct comparative validation in relevant in vivo models.

However, receptor selectivity cannot be inferred solely from ligand-binding affinity. Receptor density, heterodimer composition, membrane localization, endocytosis, and intracellular adaptor proteins all influence signaling output. Candidate therapeutics should therefore be evaluated using functional assays that measure receptor phosphorylation, dimerization, trafficking, transcriptional responses, and biological outcomes in relevant cell types.

### 6.3. Combination Therapy

NRG1 is unlikely to replace established disease-specific treatments. Its greatest clinical value may lie in combination therapy, where it could address secondary injury and repair mechanisms not targeted by current standards of care. In ischemic stroke, NRG1 could be combined with thrombolysis or mechanical thrombectomy to reduce reperfusion injury, BBB breakdown, and delayed neuroinflammation. In multiple sclerosis, NRG1-based therapy could complement immunomodulatory drugs by promoting oligodendrocyte differentiation and remyelination. In cerebral malaria, it could be administered with antimalarial treatment to reduce host-mediated neurological injury without interfering with parasite clearance. In neurodegenerative disease, NRG1 might be paired with therapies directed against amyloid, tau, or α-synuclein pathology.

Combination studies should evaluate pharmacodynamic interactions, timing, toxicity, and whether NRG1 alters the efficacy or distribution of standard therapies. Because inflammation has both protective and damaging phases, sequence of administration may be as important as dose.

### 6.4. Oncogenic NRG1 Fusions and Implications for Therapeutic Safety

A broader translational consideration for therapeutic manipulation of the NRG1-ErbB axis is the potential for unintended effects outside the CNS, including the rare but clinically important presence of oncogenic NRG1 gene fusions. In invasive mucinous adenocarcinoma of the lung, chromosomal rearrangements that place NRG1 coding sequences downstream of heterologous promoters, such as CD74, produce fusion proteins that persistently activate ErbB2/ErbB3 heterodimers and promote tumor proliferation [139,140]. Their clinical importance is underscored by the approval of zenocutuzumab, an HER2/HER3-directed bispecific antibody, for selected advanced NRG1 fusion-positive cancers [141]. Zenocutuzumab prevents ErbB3 from interacting with NRG1, leading to blocking constitutive mitogenic signaling downstream of the fusion oncoprotein.

These findings have important safety implications for therapeutic strategies targeting the NRG1–ErbB pathway in the CNS. Exogenous NRG1 administration or other approaches that broadly enhance NRG1 signaling could potentially increase ErbB2/ErbB3 activation and, in susceptible contexts, promote the growth of tumors harboring oncogenic NRG1 fusions or elevated ErbB2/ErbB3 signaling. Conversely, therapeutic inhibition of ErbB2/ErbB3, including with agents such as zenocutuzumab, could theoretically interfere with beneficial endogenous NRG1 signaling, although the consequences for NRG1-mediated neuroprotection in the CNS remain uncertain. These competing effects underscore the importance of receptor and tissue selectivity in the development of NRG1-based neuroprotective therapies. Strategies that preferentially engage ErbB4-mediated protective signaling while minimizing systemic ErbB2/ErbB3 activation may provide a more favorable therapeutic window. Development programs should therefore incorporate careful assessment of oncogenic signaling and tumor-related safety, particularly when systemic or prolonged NRG1 pathway activation is contemplated. More broadly, the potentially beneficial effects of NRG1 in neural injury and its oncogenic activity in specific molecular contexts illustrate a central theme of NRG1 biology: therapeutic benefit is likely to depend on precise, context-dependent modulation of specific receptors, cell types, and tissues rather than broad activation or inhibition of the NRG1–ErbB signaling network.

### 6.5. A Precision-Translation Framework

**Figure 5:**
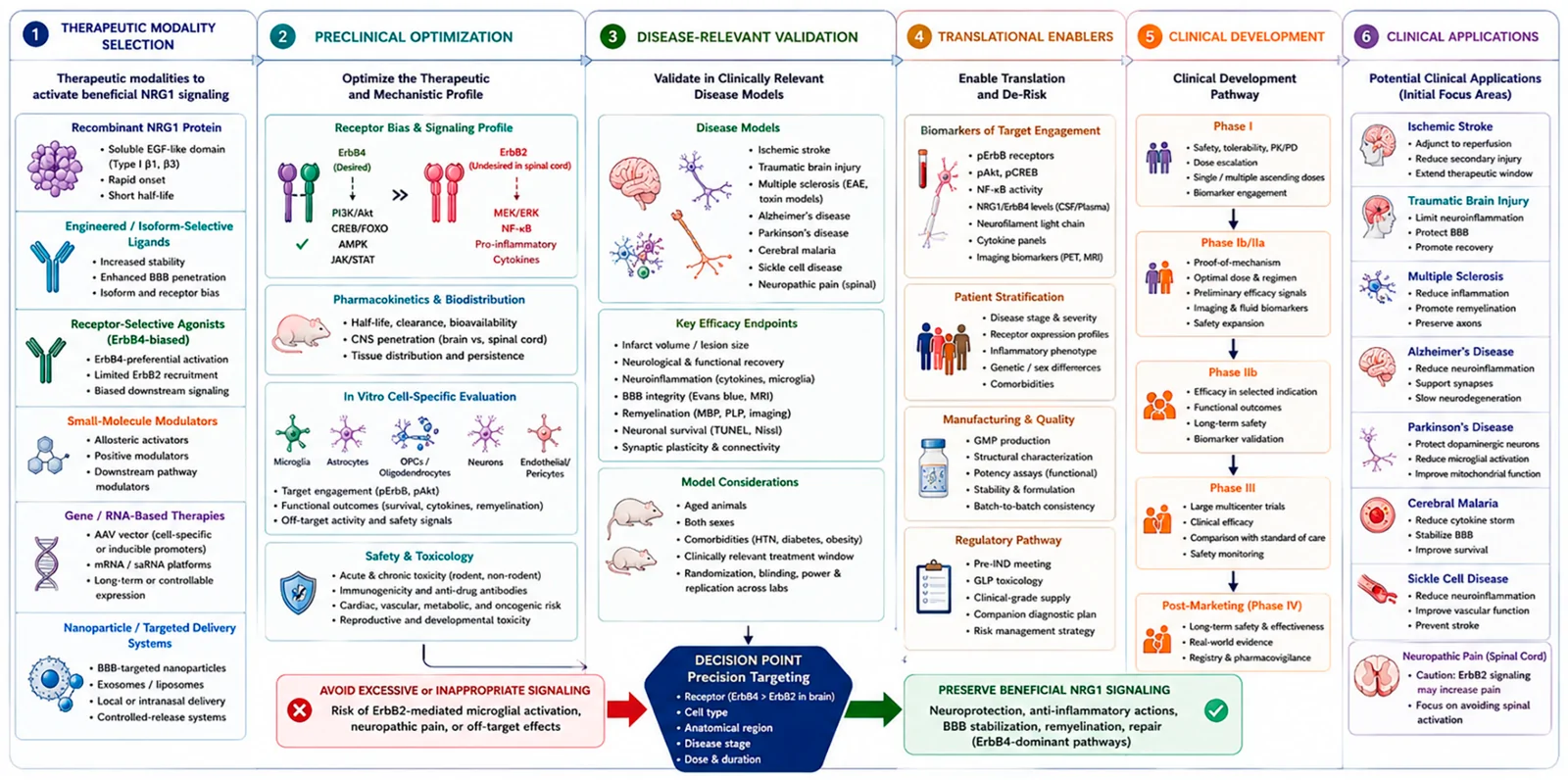
Translational development roadmap for NRG-1 based neuroimmune therapeutics.

The available evidence supports a staged translational framework for NRG1-based therapies (Figure 5). First, disease indications should be prioritized according to strength of evidence, biological plausibility, therapeutic window, and unmet need. Ischemic stroke and demyelinating disease currently have the strongest mechanistic foundations, whereas other applications require additional validation.

Second, candidate therapeutics should be selected on the basis of isoform, receptor bias, pharmacokinetics, and delivery route. Third, preclinical studies should establish cell-specific target engagement and distinguish beneficial brain signaling from potentially harmful spinal or peripheral effects. Fourth, biomarkers should be incorporated early to support dose optimization and patient selection. Finally, initial clinical trials should focus on safety, pharmacodynamics, and proof of mechanism rather than relying exclusively on broad functional outcomes. This approach shifts the field away from treating NRG1 as a generic trophic factor and toward development of a precision neuroimmune therapeutic platform.

## 7. Conclusions and Future Directions

NRG1 offers a potential therapeutic opportunity because it links inflammatory control with neurovascular protection, remyelination, and neuronal repair. However, broad activation of the NRG1–ErbB axis is unlikely to provide an optimal therapeutic strategy. The principal challenge is to preserve the coordinated benefits of NRG1 while achieving sufficient selectivity for the desired receptor complex, cell population, anatomical region, and disease stage.

The future of NRG1 therapy lies not in amplifying neurotrophic signaling indiscriminately, but in harnessing receptor-, cell-, and region-specific neuroimmune programs that simultaneously resolve inflammation and promote regeneration. This emerging precision neuroimmune framework may redefine how endogenous growth factors are translated into therapies for complex neurological disease.

Future progress will likely depend on integrating molecular neuroscience with artificial intelligence, systems biology, and precision medicine. AI-assisted analysis of multi-omics datasets, spatial transcriptomics, and computational protein engineering will facilitate the identification of receptor-specific signaling networks, optimize the design of next-generation NRG1 therapeutics, and improve biomarker-guided patient selection. Together, these advances may enable precision modulation of the NRG1–ErbB axis, allowing clinicians to selectively promote inflammatory resolution, neurovascular protection, remyelination, and neural repair while minimizing adverse effects associated with nonspecific ErbB activation.

## Data Availability

No new data were created or analyzed in this review. Data sharing is not applicable to this article.

## Abbreviations

The following abbreviations are used in this manuscript:

AD	Alzheimer’s disease
ADAM	A Disintegrin and Metalloprotease
Akt	Protein kinase B
AMPK	AMP-activated protein kinase
APOE	Apolipoprotein E
ATG5	Autophagy-related protein 5
AAV	Adeno-associated virus
BBB	Blood–brain barrier
BACE1	β-site amyloid precursor protein-cleaving enzyme 1
Bcl-2	B-cell lymphoma 2
Bcl-xL	B-cell lymphoma-extra large
BDNF	Brain-derived neurotrophic factor
CNS	Central nervous system
COX-2	Cyclooxygenase-2
CREB	cAMP response element-binding protein
CSF	Cerebrospinal fluid
DAM	Disease-associated microglia
DNA	Deoxyribonucleic acid
EAE	Experimental autoimmune encephalomyelitis
ECM	Experimental cerebral malaria
EGF	Epidermal growth factor
EGFR	Epidermal growth factor receptor (ErbB1)
ERK	Extracellular signal-regulated kinase
ErbB	Erythroblastic leukemia viral oncogene homolog receptor family
FOXO	Forkhead box O transcription factor
GABA	Gamma-aminobutyric acid
GFAP	Glial fibrillary acidic protein
GGF2	Glial growth factor 2
GSK-3β	Glycogen synthase kinase-3 beta
HO-1	Heme oxygenase-1
IL	Interleukin
IL-1β	Interleukin-1 beta
IL-6	Interleukin-6
iNOS	Inducible nitric oxide synthase
JAK	Janus kinase
MAG	Myelin-associated glycoprotein
MAPK	Mitogen-activated protein kinase
MBP	Myelin basic protein
MCAO	Middle cerebral artery occlusion
MEK	Mitogen-activated protein kinase
mRNA	Messenger RNA
MS	Multiple sclerosis
mTOR	Mammalian target of rapamycin
NF-κB	Nuclear factor-kappa B
NRG1	Neuregulin-1
OPC	Oligodendrocyte precursor cell
PD	Parkinson’s disease
PI3K	Phosphatidylinositol 3-kinase
ROS	Reactive oxygen species
RNA	Ribonucleic acid
SCI	Spinal cord injury
SCD	Sickle cell disease
STAT	Signal transducer and activator of transcription
TACE	TNF-α-converting enzyme (ADAM17)
TBI	Traumatic brain injury
TNF-α	Tumor necrosis factor-alpha
TREM2	Triggering receptor expressed on myeloid cells 2
tPA	Tissue plasminogen activator
ULK1	UNC-51-like kinase 1
ZO-1	Zonula occludens-1
